# Expression of silent mating type information regulator 2 homolog 1 and its role in human intervertebral disc cell homeostasis

**DOI:** 10.1186/ar3533

**Published:** 2011-12-08

**Authors:** Zhongying Zhang, Kenichiro Kakutani, Koichiro Maeno, Toru Takada, Takashi Yurube, Minoru Doita, Masahiro Kurosaka, Kotaro Nishida

**Affiliations:** 1 Department of Orthopaedic Surgery, Kobe University Graduate School of Medicine, 7-5-1 Kusunoki-cho, Chuo-ku, Kobe, Japan 650-0017

## Abstract

**Introduction:**

Intervertebral disc tissue homeostasis is modulated by a variety of molecules. Silent mating type information regulator 2 homolog 1 (SIRT1) plays a key role in various physiological processes. The aim of the present study was to verify the expression of SIRT1 and determine SIRT1 function in human intervertebral disc cell homeostasis.

**Methods:**

Human nucleus pulposus (NP) cells were obtained from 24 surgical patients (mean age: 39.4 years) and monolayer-cultured. SIRT1 expression was investigated using RT-PCR analysis and immunohistochemical staining. Quantitative real-time RT-PCR was performed to detect mRNA expression of *SIRT1 *and other genes: *aggrecan, collagen type 2 *and *Sox9*. The effect of SIRT1 on the extracellular matrix metabolism of NP cells was examined using recombinant human SIRT1 protein and a protein delivery reagent. Cell number and proliferation activity were measured following SIRT1 treatment. To reveal the deacetylation potential of transfected recombinant human SIRT1, western blotting for acetylated p53 was utilized. R-phycoerythrin was used for the negative control.

**Results:**

SIRT1 expression was confirmed at both mRNA and protein levels in almost all NP cells. Real-time RT-PCR analysis showed *SIRT1 *mRNA expression significantly increased with donor age (*P <*0.05, ρ = 0.492). Pfirrmann grade 3 discs showed significantly higher *SIRT1 *mRNA expression than other grades. SIRT1 treatment significantly reduced *aggrecan, Sox9 *and *collagen type 2 *mRNA expression in a dose-dependent manner in all disease classes and disc degeneration grades. Proliferation activity was decreased by SIRT1 treatment in lumbar spinal stenosis and lumbar disc herniation, Pfirrmann grade 3 and grade 4 discs. In contrast, it was significantly upregulated in idiopathic scoliosis, Pfirrmann grade 2 discs. The negative control protein did not affect extracellular matrix metabolism or proliferation activity.

**Conclusions:**

We demonstrate for the first time that SIRT1 is expressed by human NP cells. *SIRT1 *expression was significantly elevated in an early degeneration stage. SIRT1 affected both extracellular matrix metabolism and proliferation activity; the effect of SIRT1 was altered according to disease class and disc degeneration grade. SIRT1 appears to play a key role in homeostasis during the human intervertebral disc degeneration process.

## Introduction

The high morbidity of low back pain causes severe incapacity that increases medical expenses and impacts the workforce, resulting in high socioeconomic costs. Effective treatment of low back pain is therefore a matter of great public concern [1]. Although the exact pathomechanism of low back pain remains poorly understood, intervertebral disc (IVD) degeneration is thought to be a major cause [2]. Structurally, the IVD is an avascular tissue composed of an inner nucleus pulposus (NP) surrounded by an outer annulus fibrosus (AF), and sandwiched between the cartilaginous endplates of contiguous vertebral bodies. The NP cell environment is characterized by low nutrition levels, oxygen tension and pH, dependent upon diffusion across the AF and cartilaginous endplates [3-5].

IVD degeneration is considered to be a mechanically-induced and biologically-mediated pathological condition, often found concurrent with aging. With degeneration and aging, disc cells undergo substantial biological changes, including alterations in cell type, density, proliferation activity and phenotype, characterized by a compromised capability to synthesize normal matrix components and enhanced catabolic metabolism [6]. Polypeptide growth factors, such as insulin-like growth factor-1, transforming growth factor beta, and the bone morphogenetic proteins, play important roles in anabolic regulation of the IVD [7,8]. The IVD is known to express cytokines, including IL-1 and TNFα [9,10]. During aging and degeneration, the IVD undergoes extensive histomorphological changes, including fibrosis of the NP, disorganization of the AF lamellae, and thinning and calcification of the cartilaginous endplates; this results in deterioration of the IVD nutrient supply, adversely affecting disc cell viability [11,12]. Nutrient reduction for IVD cells has a powerful effect on IVD degeneration [13]. The healthy IVD is avascular, being supplied with nutrition and oxygen through passive diffusion [13]; while in the degenerating IVD, nutritional diffusion is inhibited by calcification of the endplates [14,15] and reduced blood supply from the vertebral body [16]. These changes result in decreased extracellular matrix production and increased cell apoptosis. To fully comprehend IVD degeneration, it is thus essential to determine the response of IVD cell mechanisms to reduced nutritional status and increased hypoxic conditions.

Silent information regulator 2 protein (sirtuin) is a group of nicotinamide (NAD^+^)-dependent deacetylases/ADP-ribosyltransferases initially discovered in yeast. A human homolog of silent information regulator 2, silent mating type information regulator 2 homolog 1 (SIRT1), has been reported to play a key role in transcriptional silencing, genome stability and extended lifespan following calorie restriction because of its ability to deacetylate both histone and nonhistone substrates, such as p53, NF-κB, forkhead box type O-3, and peroxisome proliferator-activated receptor-γ coactivator-1α [17,18].

SIRT1 is crucial in the pathophysiology of metabolic disease, neurodegenerative disorders, cancer, and aging [17,19]. Although the expression of SIRT1 in human IVD cells has not yet been established, SIRT1 has recently been found to be expressed by human chondrocytes and to play a key role in chondrocyte survival by regulating apoptosis, findings that could be important in the pathogenesis of osteoarthritis [20,21]. SIRT1 has also been reported to play a positive role in regulation of the expression of the cartilage-specific gene, *Sox9*, in human chondrocytes [22].

Calorie restriction was considered to be a potent inducer of SIRT1 protein expression in humans and rodents [23] and, additionally, SIRT1 levels were elevated by glucose starvation [23]. Because IVD cells must survive the harsh environmental conditions of low nutritional supply, low oxygen tension and low pH, the IVD environment is one of glucose starvation with calorie restriction. We therefore hypothesize that SIRT1 is expressed by human IVD cells to enhance survival in this harsh environment, and plays an important role in IVD cell homeostasis. The aims of the present study were to verify the expression of SIRT1 by human IVD cells, to elucidate the function of SIRT1 in IVD cell homeostasis, and to examine the relationship of SIRT1 to disc degeneration.

## Materials and methods

The collection and use of human disc specimens for the present study were approved by the ethics committee of Kobe University Hospital.

### Patients and specimens

Human NP tissues were obtained from 24 consented patients undergoing surgical procedures for lumbar spinal stenosis (LSS; *n *= 8), lumbar disc herniation (LDH, *n *= 6), or idiopathic scoliosis (*n *= 10). There were nine men and 15 women with a mean age of 39.4 years (range: 13 to 76 years). Written informed consent was obtained from all tissue donors before surgery. The degree of IVD degeneration was evaluated using preoperative magnetic resonance imaging (MRI) according to the Pfirrmann grading system, from grade 1 (normal) through grade 5 (severe degeneration) [24].

The NP was isolated after the outermost layer of the AF (about 0.5 mm) was dissected and discarded to prevent contamination with cells from surrounding ligaments. The tissue samples were first collected from the center portion of the NP and then underwent sequential enzyme digestion at 37°C with 0.025% collagenase P (Roche Applied Science, Indianapolis, IN, USA) and 0.001% deoxy-ribonuclease 2 (DNase 2; Sigma-Aldrich, St Louis, MO, USA) for 12 hours in 5% carbon dioxide and 95% air. The digested NP tissues were placed in complete tissue culture media, DMEM (Sigma-Aldrich) supplemented with 10% heat-inactivated fetal bovine serum (Sigma-Aldrich), 100 U/ml penicillin, and 100 mg/ml streptomycin. The cells were released from their matrix by centrifugation at 230 × *g *and placed in 35 mm tissue culture dishes in 5% carbon dioxide and 95% air for 3 days of preculture. For the present study, these isolated cells are defined as NP cells.

### RT-PCR analysis

Total cellular RNA was isolated from NP cells using the RNeasy Mini Kit (Qiagen, Hilden, Germany) according to the manufacturer's instructions. Reverse transcription to cDNA used 1 μg total RNA in the presence of oligo d(T) primer (Applied Biosystems, Foster City, CA, USA). The mixture was incubated at 75°C for 5 minutes and 37°C for 120 minutes.

For analysis we used 20 μl of the PCR mixture, containing 1 μl cDNA reaction product, 2 mmol/l dNTP mix, 10× PCR buffer, 25 mmol/l MgCl_2_, 5 U/μl Ampli Taq Gold (Applied Biosystems), and 1 μmol/l primers. Predesigned primers were used for *SIRT1 *(HA044263; Takarabio, Tokyo, Japan), α*_2_-macroglobulin *(HA107564; Takarabio), *cytokeratin-18 *(HA138837; Takarabio) and *CD56 *(HA106134; Takarabio) [25]. The PCR production consisted of 35 cycles of denaturing at 95°C for 1 minute, annealing at 53°C for 1 minute, and extension at 72°C for 1 minute. PCR products were separated by electrophoresis in 2% agarose gels. The gels were visualized using the LAS-3000 UV mini (FUJIFILM, Tokyo, Japan). The primer sequences are presented in Table 1.

**Table 1:** Gene-specific primer sequences used in RT-PCR and quantitative real-time RT-PCR

Gene	Primer sequence	Accession number
*SIRT1*	Forward: 5'-GCC TCA CAT GCA AGC TCT AGT GAC-3'	GenBank:NM_012238
	Reverse: 5'-TTC GAG GAT CTG TGC CAA TCA TAA-3'	
*Aggrecan*	Forward: 5'-CTA CCA GTG GAT CGG CCT GAA-3'	GenBank:NM_001135
	Reverse: 5'-CGT GCC AGA TCA TCA CCA CA-3'	
*Sox9*	Forward: 5'-GGA GAT GAA ATC TGT TCT GGG AAT G-3'	GenBank:NM_000346
	Reverse: 5'-TGA AGG TTA ACT GCT GGT GTT CTG A-3'	
*Collagen type 2*	Forward: 5'-AAG GTG CTT CTG GTC CTG CTG-3'	GenBank:NM_001844
	Reverse: 5'-GGG ATT CCA TTA GCA CCA TCT TTG-3'	
*α_2_-Macroglobulin*	Forward: 5'-TGG CGA TCG TTG ATG TGA AGA-3'	GenBank:NM_000014.4
	Reverse: 5'-CTG TCC GGC TCA CAT GGT TAG A-3'	
*Cytokeratin-18*	Forward: 5'-AGG AGT ATG AGG CCC TGC TGA A-3'	GenBank:NM_000224.2
	Reverse: 5'-TTG CAT GGA GTT GCT GCT GTC-3'	
*CD56*	Forward: 5'-TTC AAA TGC ACA GGC CCT CA-3'	GenBank:NM_000615.5
	Reverse: 5'-GTC TCA GAC ACC CAG GCT CTC A-3'	
*GAPDH*	Forward: 5'-GCA CCG TCA AGG CTG AGA AC-3'	GenBank:NM_002046
	Reverse: 5'-TGG TGA AGA CGC CAG TGG A-3'	

GAPDH, glyceraldehyde 3-phosphate dehydrogenase; SIRT1, silent mating type information regulator 2 homolog 1.

### Real-time RT-PCR analysis

The cDNA was amplified using the ABI Prism 7500 sequence detection system (Applied Biosystems). RT products were subjected to real-time PCR with the Perfect Real Time SYBR Premix Ex Taq™ (RR041A; Takarabio). Predesigned primers were used for *SIRT1, aggrecan *(HA069617; Takarabio), *Sox9 *(HA117773; Takarabio), *collagen type 2 *(HA098861; Takarabio), and *glyceraldehyde 3-phosphate dehydrogenase *(*GAPDH*) (HA067812; Takarabio). The total 20 μl volume of reaction mixture contained 10 μl SYBR Green Mix, 0.4 μl ROX dye, 6.8 μl RNase-free H_2_O, 0.8 μl primer mix and 2 μl cDNA. This PCR procedure ran for 55 denaturing cycles at 95°C for 5 minutes, then annealing and extension at 60°C for 34 seconds, according to the manufacturer's instructions. Triplicate cycle threshold (Ct) values were obtained for each sample and averaged for evaluation. The 2^-ΔΔCt ^method was then used to calculate the relative expression of each gene [26]. *GAPDH *was amplified for use as an internal control in the reaction for normalization. The primer sequences are presented in Table 1. The primer for *SIRT1 *was the same as that described for use in the RT-PCR.

### Immunohistochemical staining

The NP cells were fixed with 4% paraformaldehyde in PBS for 15 minutes at room temperature. The cells were permeabilized with 0.2% Triton X-100 in PBS for 15 minutes and blocked with 1% BSA in PBS (blocking buffer) for 30 minutes. After blocking, the cells were incubated overnight at 4°C with rabbit polyclonal anti-SIRT1 antibody (SA-427; Enzo Life Science, Plymouth Meeting, PA, USA) and mouse polyclonal anti-β-actin antibody (sc-47778; Santa Cruz Biotechnology, Santa Cruz, CA, USA) at a dilution of 1:50. The cells were then incubated with Alexa Fluor 555-conjugated anti-rabbit secondary antibody (Invitrogen, Carlsbad, CA, USA) and Alexa Fluor 488-conjugated anti-mouse secondary antibody (Invitrogen) at a dilution of 1:200 for an additional 2 hours. The cells were stained with Prolong Gold anti-fade reagent with DAPI-special packaging (Invitrogen) and observed using fluorescence microscopy (Axioskop 2 plus; Carl Zeiss, Gőttingen, Germany).

### Transfection of recombinant human SIRT1 into human nucleus pulposus cells using Pro-DeliverIN™-protein delivery reagent

Because the aim of the present study was to determine the direct effect of SIRT1 on homeostasis of human NP cells and to extrapolate possible clinical applications for SIRT1, recombinant human SIRT1 (rhSIRT1) (S8446; Sigma-Aldrich) was employed instead of SIRT1 encoding gene or siRNA, to avoid ethical issues and technical problems. A protein delivery reagent, Pro-DeliverIN™ (OZ Biosciences, Marseille Cedex, France), was used to more efficiently transfer rhSIRT1 into NP cells. Pro-DeliverIN™ is a formulation of lipids capable of capturing proteins through electrostatic and hydrophobic interactions for delivery into cells. The transfection efficiency for NP cells with Pro-DeliverIN™ was measured using rhSIRT1 labeled with the HiLyte Fluor™ 555 Labeling Kit-NH2 (Dojindo Molecular Technology, Gaithersburg, MD, USA). A microscopic visual count was performed to calculate the transfection efficiency of rhSIRT1. Cells were plated into 12-well plates at a density of 1.0 × 10^5 ^cells/well and cultured at 37°C. After 3 days of preculture, 10 μl Pro-DeliverIN™ and 10 μM labeled rhSIRT1 were mixed in a microtube and incubated for 15 minutes at room temperature. This mixture was then added to each well. The cells were incubated with the complete media at 37°C for 3 hours.

### SDS-PAGE and western blot procedure

SIRT1 is known to deacetylate p53. The deacetylation potential of transfected exogenous SIRT1 was investigated by determining the protein expression of acetylated p53 using western blot analysis. For this procedure, the exogenous fluorescent protein R-phycoerythrin, derived from cyanobacteria and eukayotic algae, was employed as the negative control. The NP cells transfected with 10 μM rhSIRT1 or R-phycoerythrin were lysed in MOPS buffer (25 mM Tris, 1% Noidet P40, 150 mM NaCl, 1.5 mM EGTA) supplemented with a protease and phosphatase inhibitor mixture (Roche Diagnostics, Basel, Switzerland) on ice for 20 minutes. The lysates were centrifuged at 19,000 × *g *to remove cell debris, the supernatants were collected, and 3× electrophoresis sample buffer (Bio-Rad, Hercules, CA, USA) was added and boiled for 5 minutes before loading onto SDS-PAGE gels. The separated proteins were transblotted onto the blotting membrane (Amersham Biosciences, Arlington Heights, IL, USA). The membranes were then probed with primary antibodies followed by incubation with horseradish peroxidase-conjugated secondary antibody. Proteins were visualized with ECL Plus reagent (Amersham Biosciences) using the chemilumino analyzer LAS-3000 mini (FUJIFLIM). Band intensities were quantified utilizing the image analysis software Image J version (NIH image; National Institutes of Health, Bethesda, MD, USA) [27]. The antibodies used in this study were rabbit anti-human acetylated p53 (Lys382) antibody (#2525S; Cell Signaling Technology, Beverly, MA, USA), rabbit anti-human p53 antibody (#9282S; Cell Signaling Technology), anti-mouse α-tubulin antibody (T9026; Sigma-Aldrich), horseradish peroxidase-conjugated goat anti-rabbit IgG and horseradish peroxidase-conjugated goat anti-mouse IgG (Amersham Biosciences).

### Effect of exogenous SIRT1 on the extracellular matrix metabolism of human nucleus pulposus cells

To investigate the effect of exogenous SIRT1 on the extracellular matrix metabolism of human NP cells, we transferred rhSIRT1 and the negative control, R-phycoerythrin, into human NP cells. After 3 days of preculture, the samples were cultured for 3 days with rhSIRT1 (0, 10 or 100 μM) or R-phycoerythrin (10 μM) in complete media and Pro-DeliverIN™ with the manufacturer's instructions. Total RNA was then isolated and real-time RT-PCR analysis was performed to evaluate the relative mRNA expression of the anabolic factors: *aggrecan, Sox9, collagen type 2 *and *GAPDH*.

### Effect of exogenous SIRT1 on cell proliferation

To examine the effect of exogenous SIRT1, cell proliferation activity was measured with the WST-8 assay using the Cell Counting Kit-8 (Dojindo Molecular Technology) following the manufacturer's instructions. The WST-8 assay is a colorimetric method in which the amount of dye generated by the activity of dehydrogenases in cells is measured. Cells from the 11 disc tissues were plated into 96-well plates (1 × 10^4 ^cells/well) and cultured in 5% carbon dioxide and 95% air at 37°C in the complete tissue culture media described above. After 3 days of preculture, rhSIRT1 (0, 10 or 100 μM) or R-phycoerythrin (10 μM) with 1 μl Pro-DeliverIN™ was added to each well. After 3 days of treatment, the number of treated NP cells in each well was calculated. Briefly, 10 μl Cell Counting Kit-8 solution was added, and the plate was incubated at 37°C for 2 hours. Absorbance at 450 nm was measured using a plate reader (Bio-Rad). The proliferative activity of the cells in each well was normalized by the cell number counted using a microscope.

### Statistical analyses

Analyses of the effects of donor age, disease and MRI grade of degeneration were assessed using the Kruskal-Wallis test. The correlation between the mRNA expression of *SIRT1 *and other genes and age was determined using the Spearman rank correlation test. Comparisons of the expression levels of *SIRT1 *mRNA among the diseases or MRI grades were analyzed using the Kruskal-Wallis test with a *post hoc *test. The effects of rhSIRT1 evaluated by changes in mRNA expression levels were assessed using the Kruskal-Wallis test with a *post hoc *test. *Post hoc *analysis was performed with the Mann-Whitney U test. All statistical analyses were performed using JMP 8 (SAS Institute, Cary, NC, USA). Statistical significance was accepted at *P <*0.05.

## Results

The analysis of donor age, disease class and the Pfirrmann disc degeneration grade found that the idiopathic scoliosis patients were significantly younger than the LDH and LSS patients, and their IVDs were categorized as grade 2 disc degeneration (mean ± standard deviation age by disc grade: grade 2, 14.4 ± 0.9 years; grade 3, 58.8 ± 12.0 years; grade 4, 54.4 ± 16.4 years; *P *< 0.01). LDH and LSS patients were similar in age and disc degeneration grade, with no significant differences seen in age or Pfirrmann grade 3 and grade 4 disc degeneration groups.

The maintenance of the character of NP cells was confirmed by data establishing that the mRNA expression of α_2_-macroglobulin, cytokeratin-18, and CD56 was maintained at the level of expression found in the primary culture (data not shown) [25].

### SIRT1 mRNA and protein expression in nucleus pulposus cells

**Figure 1:**
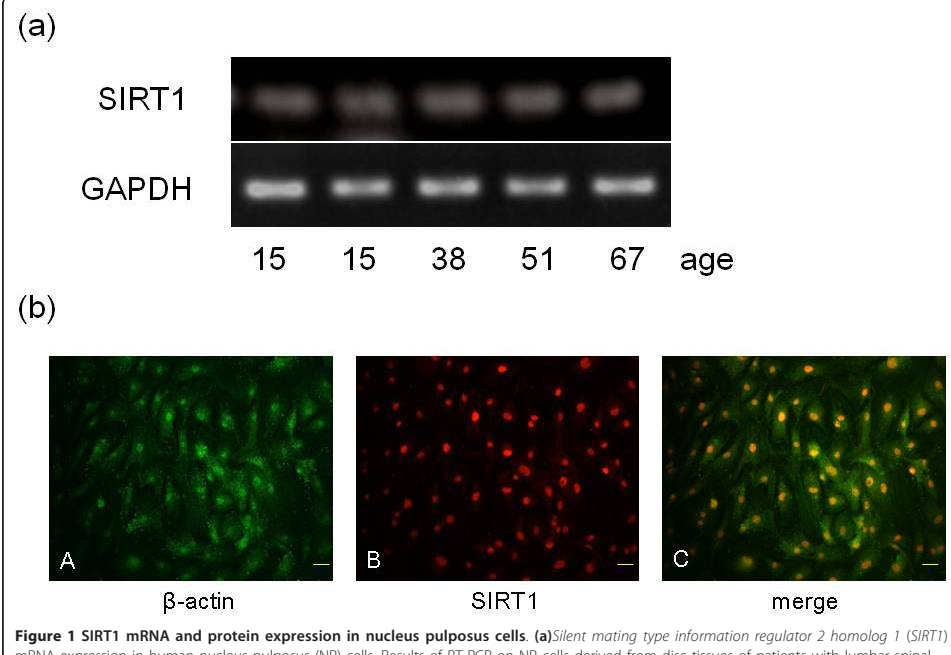
**SIRT1 mRNA and protein expression in nucleus pulposus cells**. **(a)***Silent mating type information regulator 2 homolog 1 *(*SIRT1*) mRNA expression in human nucleus pulposus (NP) cells. Results of RT-PCR on NP cells derived from disc tissues of patients with lumbar spinal stenosis, lumbar disc herniation and idiopathic scoliosis are shown. **(b) **SIRT1 protein distribution in NP cells. Immunohistochemical staining of NP cells for β-actin **(A) **and SIRT1 **(B) **(images are merged in **C**). Bars = 15 μm. GAPDH, glyceraldehyde 3-phosphate dehydrogenase.

**Figure 2:**
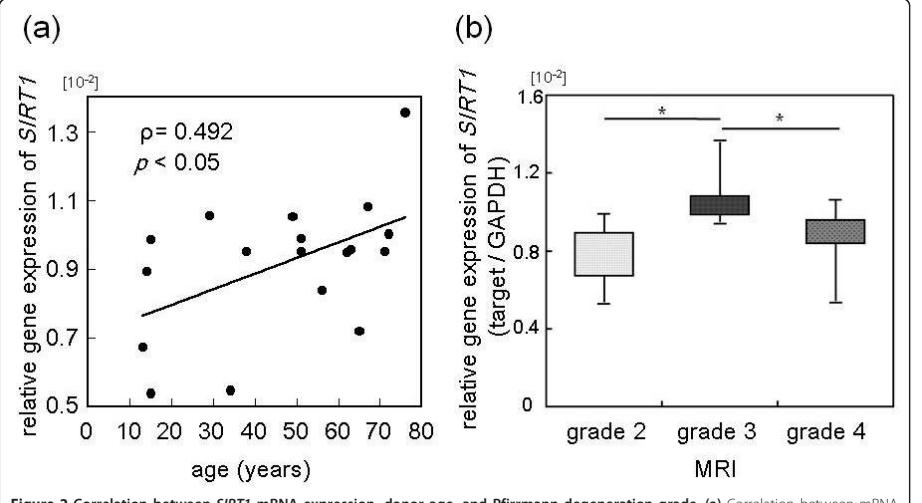
**Correlation between *SIRT1 *mRNA expression, donor age, and Pfirrmann degeneration grade**. **(a) **Correlation between mRNA expression of *silent mating type information regulator 2 homolog 1 *(*SIRT1*) and donor age in nucleus pulposus cells derived from the disc tissues of 19 patients with lumbar spinal stenosis, lumbar disc herniation, or idiopathic scoliosis. **(b) **Relationship of *SIRT1 *mRNA expression with the Pfirrmann intervertebral disc degeneration grade from magnetic resonance imaging (MRI). *P <0.05. GAPDH, glyceraldehyde 3-phosphate dehydrogenase.

RT-PCR analysis showed that *SIRT1 *mRNA was expressed in the NP cells of all donors, with expression levels varying with age (Figure 1a). In patients with idiopathic scoliosis, LDH and LSS, immunohistochemical staining confirmed SIRT1 expression and nuclear localization with β-actin localized in the cytoplasm (Figure 1b). Real-time RT-PCR results showed that *SIRT1 *mRNA expression had a significant positive correlation with age (*P *< 0.05, ρ = 0.492) (Figure 2a). There was no significant difference in *SIRT1 *mRNA expression between male and female patients (data not shown). When comparing MRI grades of all samples among the groups, the grade 3 group showed a significantly higher level of *SIRT1 *mRNA expression than the grade 2 and grade 4 groups (*P *< 0.05) (Figure 2b). No significant differences in *SIRT1 *mRNA expression were found among the idiopathic scoliosis, LDH and LSS groups.

These results confirm that SIRT1 was present in NP cells obtained from all donors at both the mRNA and protein levels. Because *SIRT1 *mRNA expression increased significantly with increasing age and was significantly higher in the Pfirrmann grade 3 disc degeneration group, we believe SIRT1 may play a role in aging and age-related chronic disorders.

### Recombinant human SIRT1 transfection into nucleus pulposus cells

**Figure 3:**
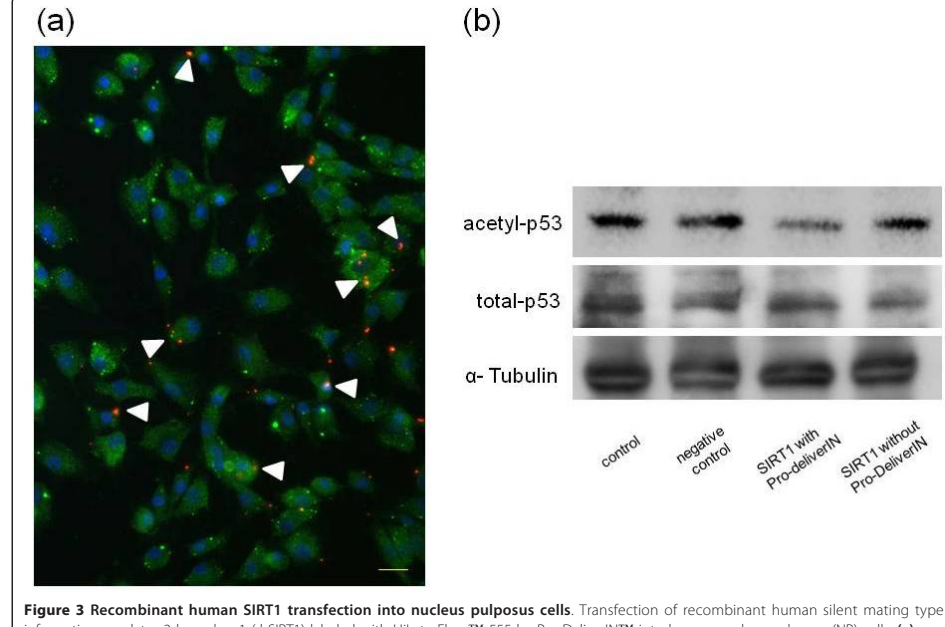
**Recombinant human SIRT1 transfection into nucleus pulposus cells**. Transfection of recombinant human silent mating type information regulator 2 homolog 1 (rhSIRT1) labeled with HiLyte Fluor™ 555 by Pro-DeliverIN™ into human nucleus pulposus (NP) cells. **(a) **Immunohistochemical staining for SIRT1, β-actin and the nucleus (4',6-diamidino-2-phenylindole). Red fluorescence shows labeled rhSIRT1 and green fluorescence shows β-actin. Arrows show labeled rhSIRT1. Of the NP cells, 37.9% were transfected with labeled rhSIRT1. Bars = 20 μm. **(b) **Western blot analysis for acetylated p53 and total p53. Western blot analysis confirmed that transfected SIRT1 deacetylated p53.

We transfected exogenous SIRT1 protein into NP cells to assess the function of SIRT1 in those cells. We first determined that rhSIRT1 was effectively transfected into NP cells by Pro-DeliverIN™ and retained its potential to deacetylate p53. Immunohistochemical staining for rhSIRT1 protein labeled with HiLyte Fluor™ 555 showed that rhSIRT1 was effectively transfected into NP cells with a transfection efficacy of 37.9%. The morphology of transfected NP cells was not changed, and the number of NP cells was not altered by transfection with rhSIRT1 (Figure 3a). Western blot revealed that transfected rhSIRT1 deacetylated p53 and did not affect total p53 and α-tubulin in NP cells (Figure 3b). The negative control protein, R-phycoerythrin, did not affect the expression levels of acetylated p53, total p53 and α-tubulin. Furthermore, the potential of rhSIRT1 to deacetylate p53 was confirmed by western blot analysis of three samples from idiopathic scoliosis, LDH and LSS patients (data not shown).

### Effect of exogenous SIRT1 on the extracellular matrix metabolism of nucleus pulposus cells

**Figure 4:**
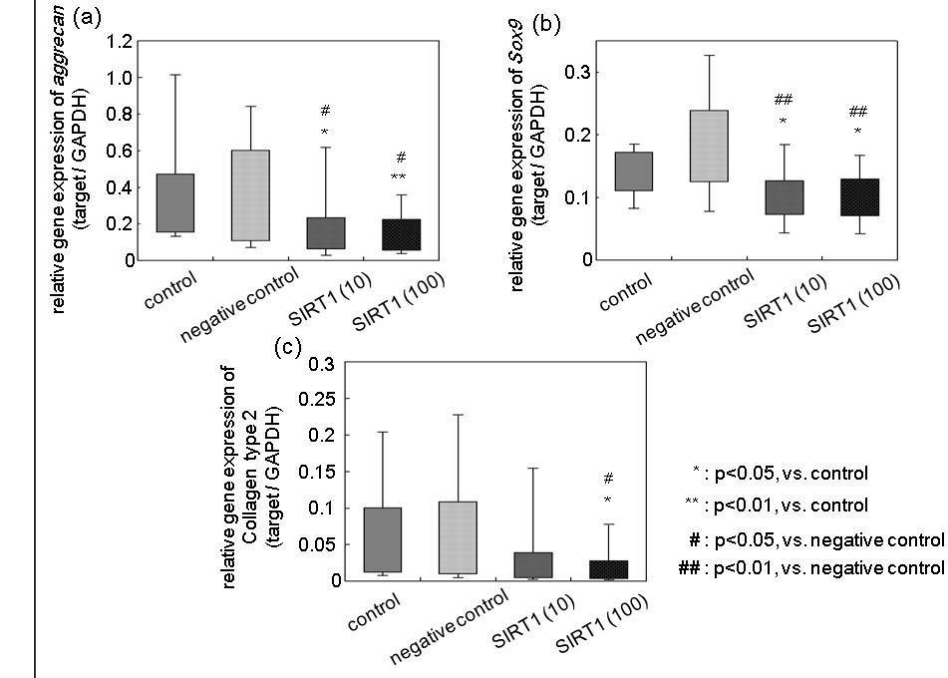
**Effect of exogenous SIRT1 on the extracellular matrix metabolism of nucleus pulposus cells**. mRNA expression of **(a) ***aggrecan*, **(b) ***Sox9 *and **(c) ***collagen type 2 *following treatment with recombinant human silent mating type information regulator 2 homolog 1 (rhSIRT1). Data were obtained from 11 patients (six lumbar spinal stenosis and lumbar disc herniation patients, and five idiopathic scoliosis patients). GAPDH, glyceraldehyde 3-phosphate dehydrogenase.

To investigate the effect of exogenous SIRT1 on the extracellular matrix metabolism of NP cells, real-time RT-PCR analysis showed that SIRT1 treatment at 10 μM and 100 μM significantly decreased *aggrecan *and *Sox9 *mRNA expression in a dose-dependent manner, compared with both the control group and the negative control group (mean values vs. control: *aggrecan *10 μM, -55.6% and 100 μM, -67%; and *Sox9 *10 μM, -35.3% and 100 μM, -41.3%) (Figure 4a, b, c). In addition, only 100 μM SIRT1 treatment significantly suppressed mRNA expression of *collagen type 2 *(mean values vs. control: 100 μM, -72.7%). The negative control, R-phycoerythrin, did not affect mRNA expression of *aggrecan, Sox9 *or *collagen type 2*. This anti-anabolic effect of exogenous SIRT1 was seen in all ages and disease classes. The comparison of the anti-anabolic effect in different MRI grades, genders and disease classes showed no significant differences and represented the same anti-anabolic effect in ECM metabolism.

### Effect of SIRT1 on the proliferation activity of nucleus pulposus cells

**Figure 5:**
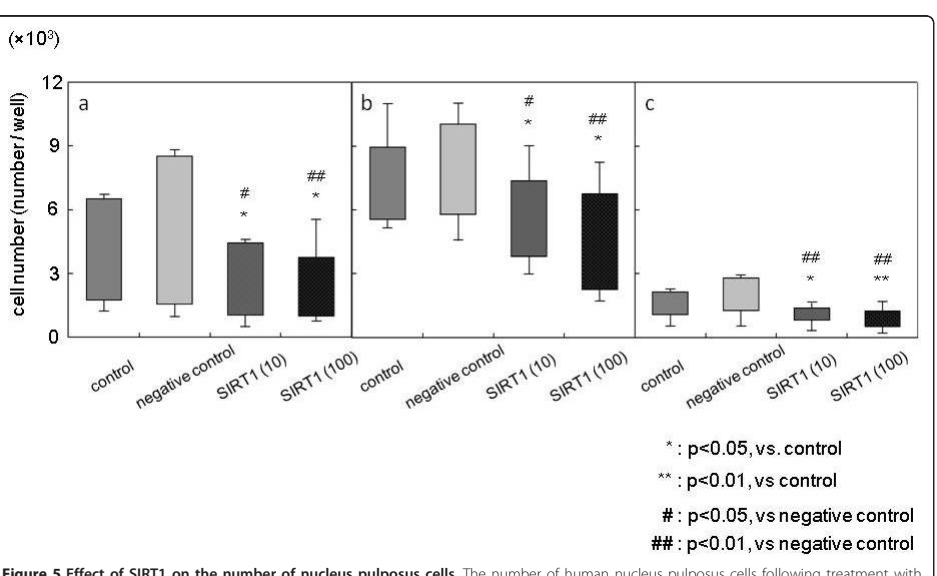
**Effect of SIRT1 on the number of nucleus pulposus cells**. The number of human nucleus pulposus cells following treatment with recombinant human silent mating type information regulator 2 homolog 1 (rhSIRT1). **(a) **Total samples (*n *= 11). **(b) **Lumbar spinal stenosis and lumbar disc herniation (*n *= 6). **(c) **Idiopathic scoliosis (*n *= 5).

The basal number of NP cells from LDH and LSS MRI grade 3 and grade 4 discs was significantly higher than that from idiopathic scoliosis MRI grade 2 discs. Treatment with both 10 μM and 100 μM SIRT1 significantly decreased the number of NP cells dose-dependently in overall samples, compared with the control and negative control groups (total: 10 μM, -34.4% and 100 μM, -40.4% (Figure 5a); LDH and LSS: 10 μM, -21.5% and 100 μM, -34.4% (Figure 5b); and idiopathic scoliosis: 10 μM, -32.3% and 100 μM, -41.5% (Figure 5c); *P *< 0.05).

**Figure 6:**
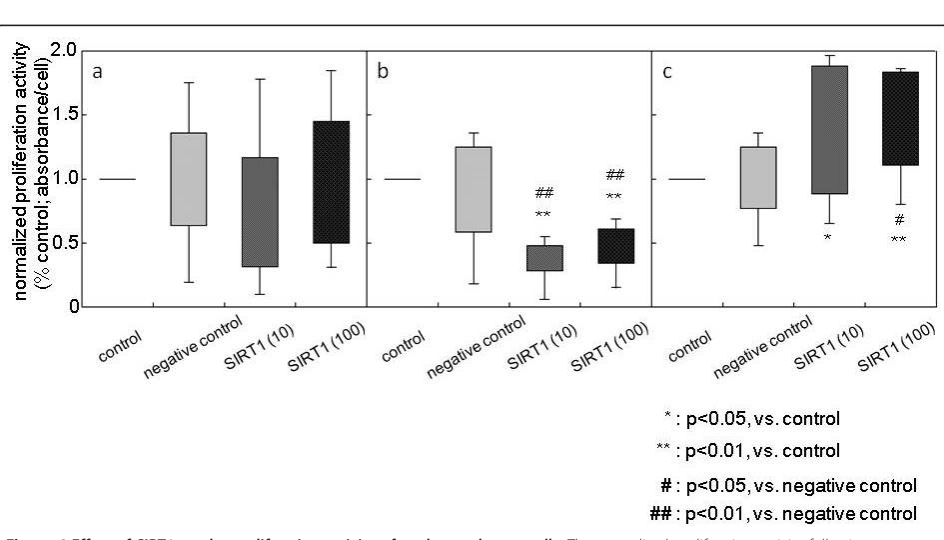
**Effect of SIRT1 on the proliferation activity of nucleus pulposus cells**. The normalized proliferation activity following treatment with recombinant human silent mating type information regulator 2 homolog 1 (rhSIRT1). **(a) **Total samples (*n *= 11). **(b) **Lumbar spinal stenosis and lumbar disc herniation (*n *= 6). **(c) **Idiopathic scoliosis (*n *= 5).

The values from the WST-8 assay of overall samples were significantly (*P *< 0.05) decreased by both 10 μM and 100 μM SIRT1 treatments (data not shown). To demonstrate the activity of individual cells, we normalized the values from the WST-8 assay to the cell number in each well, and defined this as normalized proliferation activity. The normalized proliferation activity of overall samples was not affected by treatment with SIRT1 compared with the control and negative control groups (Figure 6a). The normalized proliferation activity of LDH and LSS samples was significantly decreased by both treatments of 10 μM and 100 μM SIRT1 (vs. control as percentage of control: 10 μM, -59.4% and 100 μM, -46.4%; both *P *< 0.01) (Figure 6b). In contrast, in idiopathic scoliosis samples this anti-proliferation activity was reversed, with SIRT1 treatment significantly upregulating the normalized proliferation activity (vs. control as percentage of control: 10 μM, +37.5% (*P *< 0.05) and 100 μM, +60.6% (*P *< 0.01)) (Figure 6c). The negative control, R-phycoerythin, did not affect either the number or normalized proliferation activity of NP cells.

## Discussion

Our study demonstrates for the first time that SIRT1 is expressed by human NP cells. Almost all NP cells express SIRT1 protein regardless of donor age. Furthermore, *SIRT1 *mRNA expression levels significantly increased with advancing age. It has been suggested that the effects of SIRT1 vary during cellular events. SIRT1 can regulate endothelial nitric oxide to protect against cardiovascular disease, exert a cardioprotective role in heart failure [28], and protect against neurodegenerative pathological changes [17]. Although SIRT1 does not always play a positive role in a variety of physiological processes and pathophysiology, Li and colleagues found that SIRT1 inhibition increased the resistance of mammalian cells to oxidative stress [29]. SIRT1 also enhanced neural survival in acute anoxic injury and provided neural protection [30,31].

Conflicting findings on the effects of SIRT1 have been reported and the functions of SIRT1 are still unclear. The function of SIRT1 in the homeostasis of human IVD tissues has also not been clarified. A few investigators have reported the function of SIRT1 in articular chondrocytes. Takayama and colleagues determined that SIRT1 regulated apoptosis in human chondrocytes through modulation of mitochondria-related apoptotic signals [20]. Dvir-Ginzberg and colleagues found that elevation of SIRT1 protein levels in human chondrocytes led to a dramatic increase in the cartilage-specific gene expression of *aggrecan, collagen type 2 *and *collagen type 9*, while a reduction in SIRT1 levels or activity significantly lowered the expression of these genes [22]. These two reports suggest that SIRT1 plays a positive role in the maintenance of chondrocyte homeostasis, the loss of which may lead to the development of osteoarthritis [32]. In our study, however, SIRT1 treatment significantly decreased the expression of *aggrecan, Sox9 *and *collagen type 2 *mRNA and the proliferation activity was downregulated in degenerated IVD tissues. Transfection with rhSIRT1 thus seemed to have no positive effect on anabolism. This difference in function of SIRT1 in extracellular matrix metabolism may relate to differences in the Pasteur effect in articular chondrocytes and IVD cells. While glucose is the main energy resource for both articular chondrocytes and NP cells, these cells differ in glucose metabolism under anoxic stress. Under aerobic conditions, articular cartilage cells show a negative Pasteur effect, but under anoxic conditions both glucose uptake and lactate production are greatly inhibited [33]. IVD cells, on the other hand, show a positive progressive Pasteur effect [34]. Under lowered oxygen conditions, IVD cells show reduced oxygen consumption but increased lactate production. The metabolism of NP cells depends primarily on glycolysis, unlike articular chondrocytes. Consequently, NP cells are rich in NAD^+ ^produced by glycolysis [35]. Increased NAD^+ ^levels may accelerate the NAD^+^-dependent deacetylase function of SIRT1. The difference in NAD^+ ^production may explain the different functioning of SIRT1 in articular chondrocytes and IVD cells.

In our study, *SIRT1 *mRNA expression showed different trends in aging and degeneration. *SIRT1 *mRNA expression was significantly higher with increasing age, whereas NP cells from MRI grade 3 degeneration discs had higher *SIRT1 *mRNA expression than did grades 2 and 4. Although the pathomechanism of disc degeneration remains unclear, no relationship between age and macroscopic IVD degeneration was found in a postmortem study of 273 cadavers [36]. Sowa and colleagues reported finding different gene expressions of NP cells between aging and degeneration in their rabbit model [37]. If SIRT1 is one of the constitutive inducers of human IVD degeneration, grade 4 degeneration discs should show stronger *SIRT1 *mRNA expression than lower grade discs.

Kauppila used angiograms of cadaveric lumbar spines to show that vascular changes occurred prior to disc degeneration and that disturbance in the nutrient supply preceded early disc degeneration [38]. Miller and colleagues stated that mechanical stress and the nutrition pathway were responsible for early disc degeneration found in their study [36]. From these reports, the deterioration of the internal environment of IVD tissues, caused by disturbance of the nutrient supply, was thought to progressively decrease synthesis, leading the IVD into early stages of degeneration. As discussed above, calorie restriction is thought to be a strong inducer of SIRT1 protein expression in humans and rodents [23]. SIRT1 might be induced in the early disc degeneration stage when the disc internal environment begins to deteriorate from a disturbance in the nutrient supply. The deterioration of the internal environment prior to early disc degeneration may thus induce SIRT1 expression. The decreasing oxygen tension in advanced IVD degeneration may also affect the function of SIRT1 on proliferation activity by a positive Pasteur effect.

The adequacy of the nutrition supply is also dependent on cell activity. Masuda and colleagues have stated that proliferating cells stimulated by growth factors require more nutrition [39]. Unless the nutrition supply is adequate to support their activity, proliferating cells die [39]. Indeed, increased IVD cell proliferation and density are so commonly seen around a tissue cleft in degenerative discs that one may assume neovascularization contributes to improving the nutrition supply and stimulating cell proliferation in areas adjacent to the newly formed blood vessels. Consequently, increased cell proliferation has been used as an indicator for disc degeneration [6,40]. Furthermore, Boos and colleagues reported that cell death was increased in degenerated discs [41] and apoptosis was recently reported to play an important role in disc degeneration [42]. Adaptation to the deteriorating internal environment associated with inadequate nutrient supply may possibly reduce the anabolic activity, cell number and proliferative activity of NP cells.

Based on our results, especially the finding that the effect of SIRT1 on proliferation activity is altered by advanced IVD degeneration, the authors postulate that SIRT1 plays a role in the maintenance of IVD tissue homeostasis. Namely, SIRT1 plays a positive role for IVD proliferation in MRI grade 2 discs and is then upregulated in grade 3 discs, where its role seems to be to suppress ECM metabolism and proliferation activity in IVD tissues after grade 3 degeneration. SIRT1 might therefore be upregulated at an early stage of degeneration, causing NP cells to downregulate both anabolic and proliferation activity, thus helping NP cells survive the deteriorating internal environment resulting from preceding vascular changes. SIRT1 was initially upregulated to adapt to deteriorating internal circumstance in early degeneration, but the failure of NP cells to adapt resulted in decreased *SIRT1 *mRNA expression in advanced degeneration.

In the present study, the samples were obtained from patients but cultured under normal conditions (5% carbon dioxide, rich nutrition and normal pH), different from the *in vivo *environment. Consequently, our results may reflect the function of SIRT1 under the culture conditions compared with the limited *in vivo *environment in which the tissues exist. Further research is needed to elucidate the function of SIRT1 in human IVD degeneration.

## Conclusions

We have demonstrated that SIRT1 is expressed by human NP cells. *SIRT1 *mRNA expression increased significantly with donor age and was seen most strongly in early grade 3 disc degeneration. Treatment with exogenous SIRT1 reduced anabolic activity in NP cells. In addition, proliferation activity was upregulated by SIRT1 treatment in idiopathic scoliosis samples with relatively nondegenerated tissues, and was downregulated in LDH and LSS samples with more degenerated IVD tissues. These results are interpreted to indicate that SIRT1 participates in the degenerative changes in extracellular matrix metabolism as an early responder to IVD degeneration rather than a constitutive inducer of it. SIRT1 may be upregulated to induce the downregulation of anabolic and proliferation activity of NP cells to aid survival in the deterioration of the disc environment arising from preceding vascular changes. We suggest that SIRT1 may play a key role in the early stage of disc degeneration in human IVD tissues. The study of SIRT1 function in NP cells may lead to new approaches to study the pathomechanism of IVD degeneration.

## Abbreviations

AF: annulus fibrosus; BSA: bovine serum albumin; DMEM: Dulbecco's modified Eagle's medium; GAPDH: glyceraldehyde 3-phosphate dehydrogenase; IL: interleukin; IVD: intervertebral disc; LDH: lumbar disc herniation; LSS: lumbar spinal stenosis; MRI: magnetic resonance imaging; NAD^+^: nicotinamide; NF: nuclear factor; NP: nucleus pulposus; PBS: phosphate-buffered saline; PCR: polymerase chain reaction; rhSIRT1: recombinant human silent mating type information regulator 2 homolog 1; RT: reverse transcription; siRNA: small interfering RNA; SIRT1: silent mating type information regulator 2 homolog 1; TNF: tumor necrosis factor.

## Competing interests

The authors declare that they have no competing interests.

## Authors' contributions

ZZ conceived the study and made substantial contributions to the study design and to writing the manuscript. KK also participated in the conception and design of the study, secured funding and cowrote the manuscript. KM participated in the design of the study, conducting the experiments and performed the statistical analysis. TT interpreted data and contributed to draft the final manuscript. TY also acquired and interpreted data and helped with drafting the manuscript. MD helped to secure funding and contributed to the design and conception of the study. MK coordinated the study and contributed to draft the final manuscript. KN participated in the design of the study and finalized the manuscript. All authors read and approved the final manuscript.
